# Supplementary material for: CRMP2 as a Candidate Target to Interfere with Lung Cancer Cell Migration
Source: Biomolecules. 2021 Oct 18;11(10):1533. doi: 10.3390/biom11101533 (PMC8533992; doi:10.3390/biom11101533)
Supplement: Supplementary file 1 [file biomolecules-11-01533-s001.zip › biomolecules-1382511-supplementary.pdf]

(Morales X, et al.)

SUPPLEMENTARY METHODS

Cell viability assay

H1299 and A549 cell viability was evaluated using the neutral red assay. To that aim, 5×10^3 cells were plated in 96-well plates (Sigma-Aldrich, Steinheim, Germany) in complete RPMI culture media. After 24-, 48-, and 72 h of culture, the medium was removed, and the cell viability was tested as previously described by Irigoyen et al.[18]. Three independent plates were quantified, in which four wells were analyzed per cell type and condition (number of wells, n = 12). Data were normalized relative to the non-transfected A549 and H1299 cell lines at each time point.

Centromere polarization

To assess centromere polarization after CCL21 stimulation, 5×10^4 cells were seeded on 8-well slides (Lab-Teck, Roskilde, Denmark) covered with 50 μg/ml of collagen type I (BD Biosciences, San José, CA, USA) and were grown to confluence. The monolayer was scraped off with a pipette tip as described in the kinetic parameters section. After 3 h, cells were fixed and stained with anti-tubulin and anti-γ-tubulin antibodies as described in the confocal microscopy imaging section.

The centromere polarization was scored as oriented when the γ-tubulin signal was in the sector (90°) containing the leading edge. Data are shown as the percentage of cells with an oriented centromere. Three independent replicas, in which six fields, and ten cells per field were randomly analyzed (n = 180).

2D invadopodia gelatin degradation assay

To study ECM remodeling, 8-well slides (Lab-Tek, Roskilde, Denmark) were coated with 50 μg/ml poly-D-lysine for 15 min, washed with PBS, and cross-linked for 15 min with 0.5% glutaraldehyde. Afterward, wells were washed with PBS, and 100 μl of pigskin gelatin conjugated with Oregon Green 488 (Life Technologies, Barcelona, Spain) (1 mg/ml) were added and incubated for 15 min at room temperature. After gelling, the fluorescent signal was quenched with 5 mg/ml sodium borohydride for 3 min at room temperature. Subsequently, 100 cells/μl were seeded on cross-linked fluorescent gelatin in RMPI culture media supplemented with 10% serum. After 48 h, cells were fixed with 4% PFA and images were acquired as described in the confocal microscopy imaging section. Proteolytic activity was inhibited or stimulated by incubating with GM6001 (25 μM) and EGF (5 ng/ml), respectively, as described in the cell treatments section.

The percentage of cells showing degradation was quantified using the ImageJ software. Invadopodia were identified as actin-rich protrusions that co-localized with matrix degradations (data not shown). Three gels of each cell type and condition were analyzed, in which 50 cells were randomly acquired (Number of cells, n = 150).

SUPPLEMENTARY FIGURES

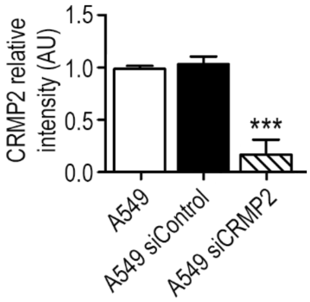

Supplementary Figure S1. Quantification of CRMP2 expression levels from total protein extracts shown in (**Figure 1A**) using the ImageJ software (Arbitrary Units, AU). Each sample was normalized to β-Actin expression and, subsequently, to the expression of CRMP2 in the A549 control cell line. *** (p<0.001).

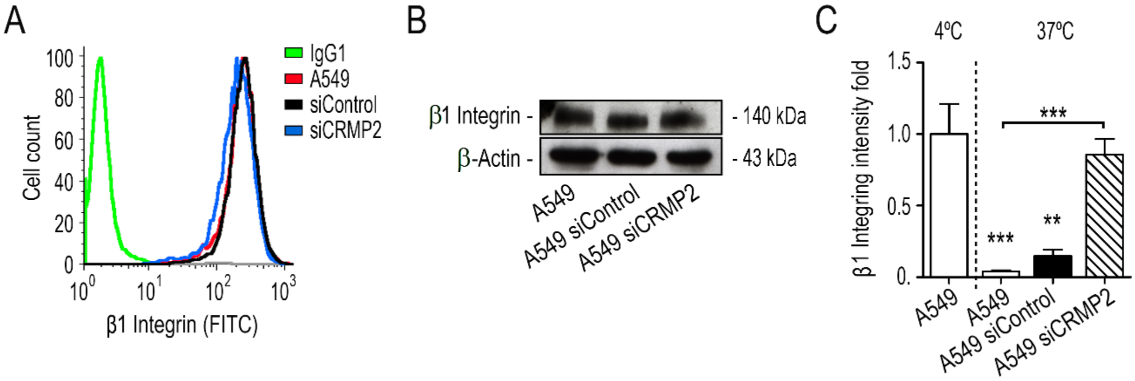

Supplementary Figure S2. (**A**) Flow cytometry and (**B**) Western blot analysis of β1 integrin expression in wild type and A549 cells with CRMP2 transfected with specific siRNAs targeting CRMP2 expression or non-target siRNA sequences (25 μM). β-Actin was used as a Western blot loading control. (Number of blots, n = 3); (**C**) quantification of the Mean Fluorescence Intensity (MFI) of β1 Integrin (Arbitrary Units, AU) on the cell surface from the images shown in (**Figure 2C**). Data are shown as a fold ratio over the intensity of β1 integrin at the cell surface in the control cell line A549 at 4 °C. *** (p<0.001). ** (p<0.01).

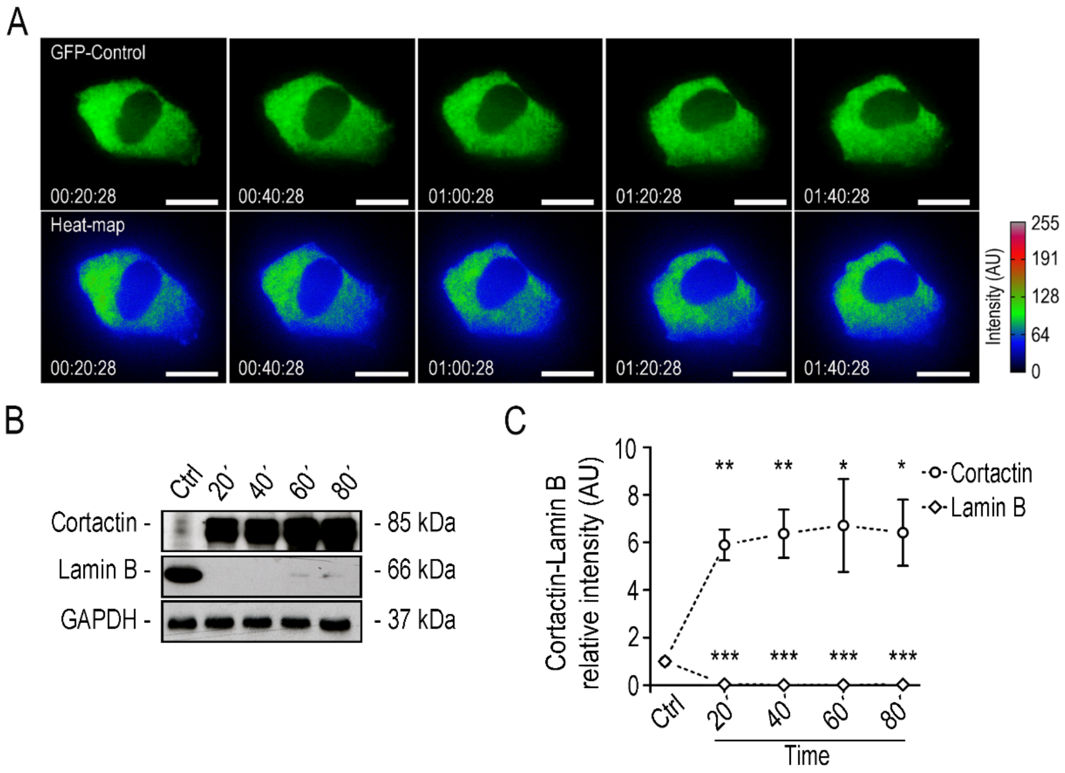

Supplementary Figure S3. (**A**) Representative confocal images and heat map of cytoplasmic GFP-empty vector distribution in A549 cells after CCL21 stimulation (200 ng/ml) at indicated time points. Cell migration was captured by confocal video-microscopy every 2 min for 2 h. The GFP heat map was rendered using the ImageJ software. Scale bar = 20 microns. (Number of videos, n = 10); **B**) quantification of Cortactin and Lamin B expression levels from total protein extracts using the ImageJ software (Arbitrary Units, AU). Each sample was normalized to β-Actin expression and, subsequently, to the expression of Cortactin and Lamin B in the A549 control cell line; (**C**) quantification of Cortactin and Lamin B expression levels from total protein extracts shown in (**B**) using the ImageJ software (Arbitrary Units, AU). Each sample was normalized to β-Actin expression and, subsequently, to the expression of Cortactin and Lamin B in the A549 control cell line. *** ($p<0.001$). ** ($p<0.01$). * ($p<0.05$).

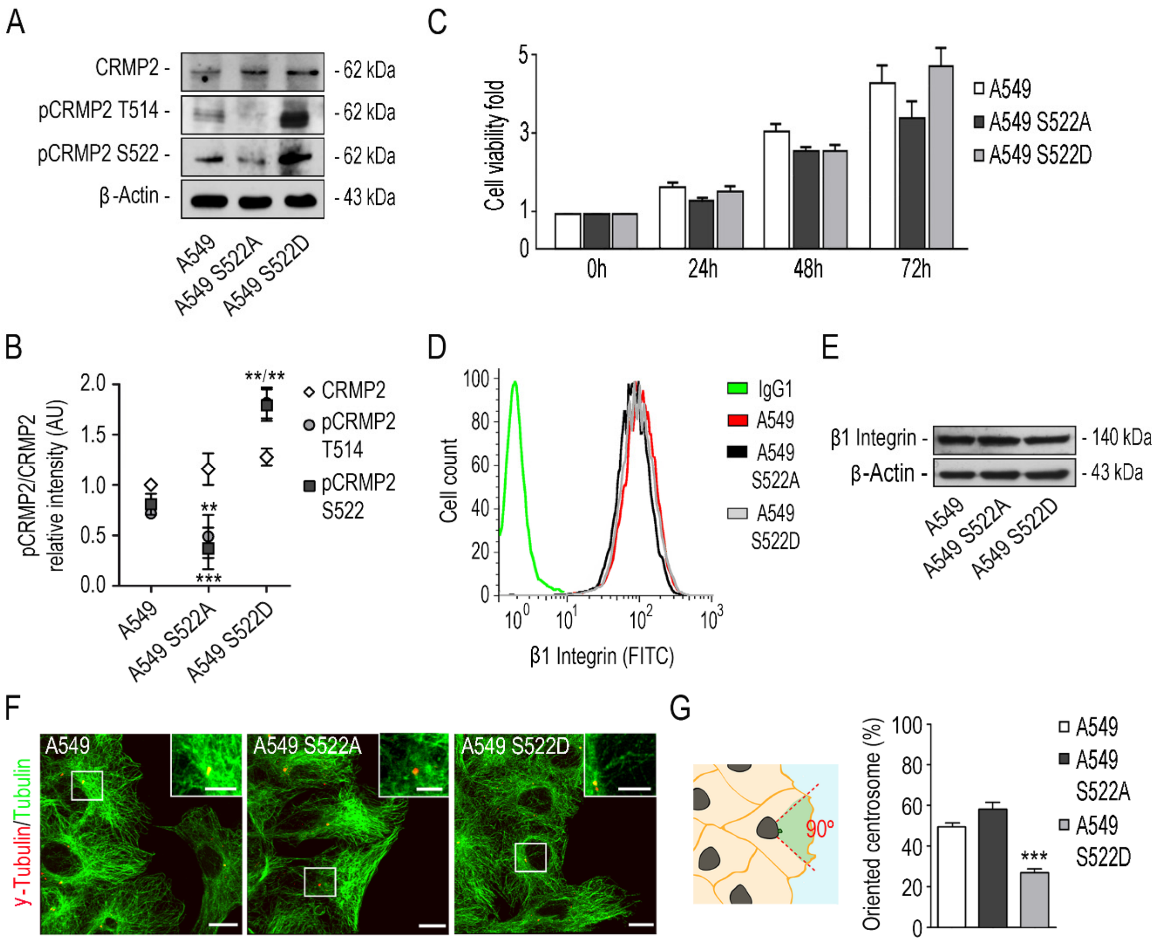

Supplementary Figure S4. (**A**) Western blot analysis of CRMP2 and pCRMP2 (T514 and S522) expression in the control cell line A549 and the A549 S522A phosphodefective and A549 S522D phosphomimetic mutants more than ten cell passages after transfection and selection with G418. β-Actin was used as a Western blot loading control. (Number of blots, n = 3); **B**) quantification of CRMP2 and pCRMP2 (T514 and S522) expression levels from total protein extracts shown in (**A**) using the ImageJ software (Arbitrary Units, AU). Each sample was normalized to β-Actin expression and, subsequently, to the expression of CRMP2 in the A549 control cell line; (**C**) cell viability in the control cell line A549 and the A549 S522A phosphodefective and A549 S522D phosphomimetic mutants at the indicated time points (0-, 24-, 48-, and 72 h). Data are shown as a fold ratio over wild-type A549 cells. (Number of wells, n = 12); (**D**) flow cytometry analysis of β1 integrin expression at the cell surface in the wild-type A549 and pCRMP2 S522 mutants. β1 integrin expression was assessed at 4 ºC in serum-free media. (n = 3); (**E**) western blot analysis of β1 integrin expression in the same cells. β-Actin was used as a Western blot loading control. (Number of blots, n = 3); (**F**) tubulin (green) and γ-tubulin (red) detection by immunofluorescence as detected by confocal microscopy in wild-type A549 and pCRMP2 S522 mutants after CCL21 stimulation (200 ng/ml). Images show maximum intensity projections of confocal images. Scale bar = 10 microns. White insets show enlarged areas of centromere position. Scale bar = 5 microns; (**G**) drawing shows a schematic representation of centromere polarization in migrating cells. The right panel presents the percentage of cells with polarized centromeres after CCL21 stimulation. (Number of cells, n = 180). *** ($p<0.001$). ** ($p<0.01$).

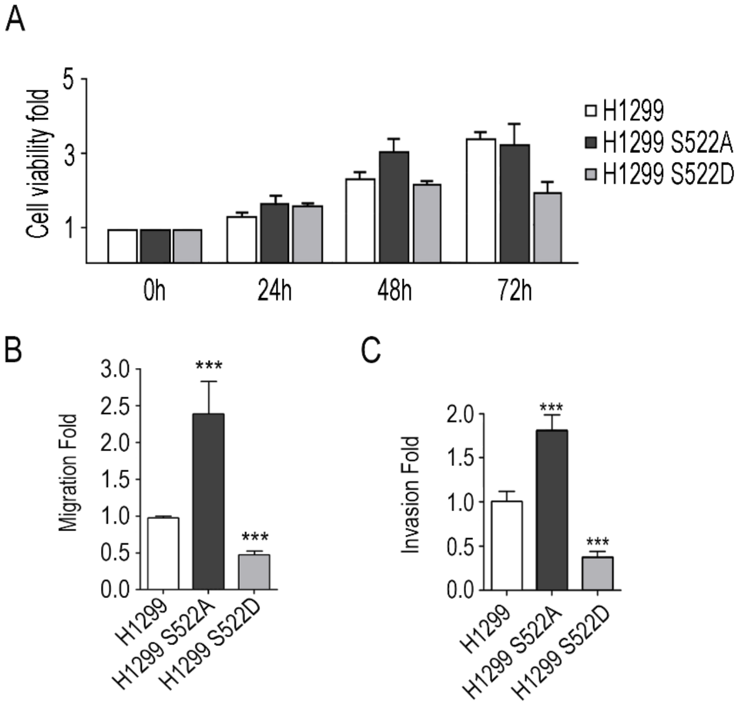

Supplementary Figure S5. (**A**) Cell viability in the control cell line H1299 and the H1299 pCRMP2 S522 mutants at the indicated time points (0-, 24-, 48-, and 72 h). Data are shown as a fold ratio over wild-type H1299 cells. (Number of wells, n = 12); (**B**) boyden migration assays to 20% serum of the same cell lines. Data are shown as a fold ratio over wild-type H1299 cells. (Number of images, n = 12); (**C**) invasion assays across Matrigel covered Boyden chambers to 20% serum. Data are shown as a fold ratio over wild-type H1299 cells. (Number of images, n = 12). *** ($p<0.001$).

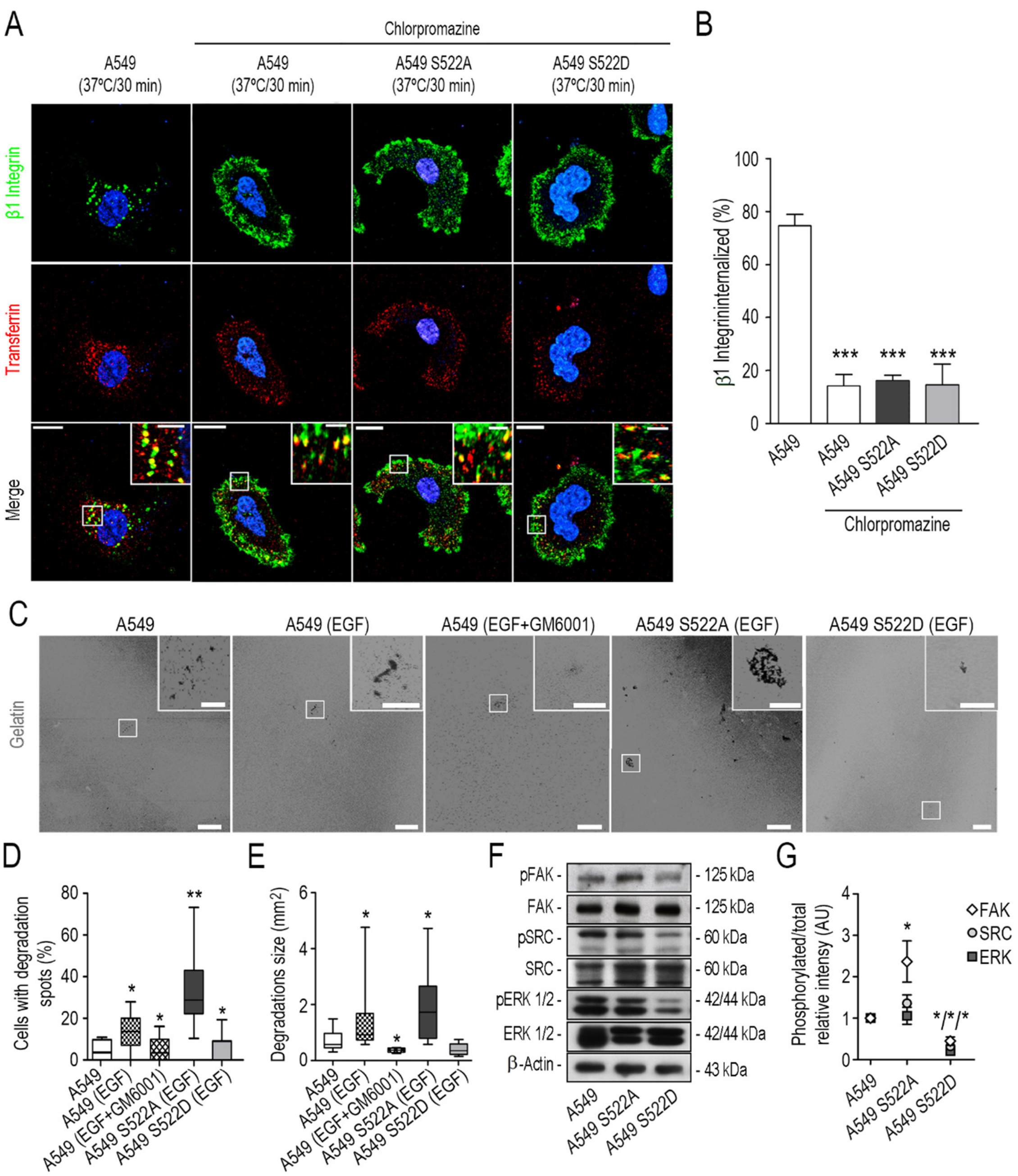

Supplementary Figure S6. (**A**) Representative maximum intensity projections confocal images showing β1 integrin (green), transferrin (red), and DRAQ5 (blue) staining in wild type A549 or the A549 pCRMP2 S522 mutants. Cell surface receptors uptake was stimulated for 30 min at 37 °C in serum-free media. Vesicle internalization was blocked by treatment with chlorpromazine (50 μM) at 4 °C. Scale bar = 15 microns. White insets show enlarged areas with strong β1 integrin and transferrin co-localization. Scale bar = 5 microns; (**B**) quantification of β1 integrin uptake by flow cytometry in the wild-type A549 and pCRMP2 S522 mutants treated or not with chlorpromazine (50 μM). Data are shown as the percentage of internalized β1 integrin relative to membrane expression at 4 °C. (n = 3); (**C**) representative maximum intensity of invadopodia degradation areas (black dots) by confocal imaging of A549 control and pCRMP2 S522 mutants seeded on fluorescence-conjugated gelatin (grey background). As a positive control, proteolytic activity was stimulated with EGF (5 ng/ml). Negative control was obtained by treatment with the MMPs inhibitor GM6001 (25 μM). Scale bar = 15 microns. White insets show enlarged areas of proteolytic activity (quenched gelatin). Scale bar = 5 microns; (**D**) quantification of the percentage of cells that presented degradation spots from (**C**); (**E**) average size of the degradation spots obtained from each experimental group. (Number of cells, n = 150); (**F**) western blot analysis of the expression of integrin signaling molecules in total

cytoplasmic protein extracts obtained from migrating wild-type A549 cells or pCRMP2 S522 mutants. β-Actin was used as a Western blot loading control. (Number of blots, n = 3); (**G**) quantification of total and phosphorylated expression levels of FAK, Src, and ERK kinases from protein extracts shown in (**F**) using ImageJ software (Arbitrary Units, AU). Each sample was normalized to β-Actin expression and, subsequently, to the expression of FAK, Src, and ERK in the A549 control cell line. *** ($p<0.001$). ** ($p<0.01$). * ($p<0.05$).

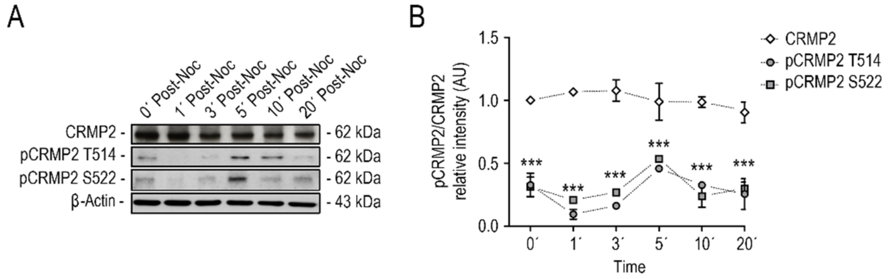

Supplementary Figure S7. (**A**) Western blot analysis of CRMP2, pCRMP2 T514, and S522 expression in A549 cells after nocodazole (10 μM, 4 h) removal and serum stimulation at the indicated time points (0-, 1-, 3-, 5-, 10-, and 20 min). β-Actin was used as a Western blot loading control. (Number of blots, n = 3); (**B**) quantification of CRMP2, pCRMP2 T514, and pCRMP2 S522 expression levels shown in (**A**) using the ImageJ software. Each sample was normalized to β-Actin expression and, subsequently, to CRMP2 expression. *** ($p<0.001$).
